# Estimating the minimum control count of random network models

**DOI:** 10.1038/srep19818

**Published:** 2016-01-28

**Authors:** Derek Ruths, Justin Ruths

**Affiliations:** 1 School of Computer Science, McGill University, Montreal, Canada; 2 Engineering Systems and Design, Singapore University of Technology and Design, Singapore

## Abstract

The study of controllability of complex networks has introduced the minimum number of controls required for full controllability as a new network measure of interest. This network measure, like many others, is non-trivial to compute. As a result, establishing the significance of minimum control counts (MCCs) in real networks using random network null models is expensive. Here we derive analytic estimates for the expected MCCs of networks drawn from three commonly-used random network models. Our estimates show good agreement with exact control counts. Furthermore, the analytic expressions we derive offer insights into the structures within each random network model that induce the need for controls.

Recent advances in applying techniques from control theory to network science have provided new conceptual perspectives and quantitative approaches on complex networks. In particular, the question of controllability — the problem of driving the state of a system over time to a desired endpoint — has yielded new ways of thinking about the relationship between network structure and function^1^,^2^. When the dynamical system evolving on a complex network is linear, structural controllability provides, among other things, a way of using only the network structure to determine the minimum number of independent inputs must be attached to a network in order to control the entire system, which here we call the *minimum control count* or MCC^1^,^3^,^4^.

Because these and other control-theoretic constructs relate to the propagation of influence through a system, it is not surprising that the measure has been shown to take on a range of values depending on the system of interest^1^. Recent work has shown that a particular decomposition of the minimum control count, called the *control profile*, separates real-world networks into clearly defined classes that have conceptual and functional significance^2^. This demonstrates that the minimum control count is a statistic that provides functional insight that can be used to compare networks, regardless of whether the controls are, themselves, complete or feasible. It is worth noting that this line of research of network science using structural control analysis results is distinct from parallel (and complementary) efforts to determine the feasibility of the input signals required to drive the network between desired states, which is a question of control synthesis and implementation^5^,^6^,^7^.

As a result, the minimum control count has joined a pantheon of existing network measures, including variants of centrality, clustering, motifs, network diameter, and degree distribution. All of these have been used to better understand the structure and behavior of a wide array of complex systems (e.g.,^8^,^9^). Among such studies, it is standard to employ null network models as a way of establishing the significance of a particular measure of interest (e.g.,^10^,^11^). These null models have typically been implemented as ensembles of networks generated under a specific random network model. As a result, establishing the expected distributions of network properties of random network models has been and continues to be a theoretical enterprise with important practical implications.

At their most basic level, random network models are described by well-defined generative processes. These processes make it possible, in some cases, to derive exact or approximate analytic expressions for particular network features, such as diameter, degree distribution, and clustering^12^,^13^. Because analytic expressions are computationally trivial, such analytic forms make the analysis of otherwise expensive-to-compute network properties tractable (e.g., motif identification and diameter).

The minimum control count can be computed in *O*(*V*^2^log*V*) time by finding a maximum matching of the network^14^. Thus, the algorithm is, technically, tractable. However, for networks of any appreciable size the computation is expensive.

In this paper, we provide an attractive and theoretically informative alternative to brute-force computing the MCC for well-known random network models. Here we derive tight analytic estimates for the MCC in three of the most commonly-used random network models: Erdos-Renyi, Barabasi-Albert, and local attachment^15^,^16^,^17^. Our estimates show extremely good agreement with empirical results, indicating that they can be used as a computationally efficient and reliable way of determining the MCC of these random network models. Furthermore, our derivations explicitly show the component-wise composition of the minimum control count in each random network model.

## Controllability

A system is controllable if it is possible to drive the system from any initial state to any desired final state in finite time through the application of a time-varying input or set of inputs. Controllability is an important property of systems of interest because it indicates the feasibility and complexity of arbitrarily influencing the state of the systems.

Due to their sheer size and complexity, networks offer a distinct and novel challenge to the task of determining control-related properties. Many of the existing methods in the control theory literature do not scale efficiently to large networks. Moreover, networks present completely new questions to be addressed. The first of these seeks to determine which (and how many) nodes in a network must receive direct external control in order to render the system controllable.

Prior work in the control of real-world, directed networks has focused on answering this question^1^,^18^. This body of work considers a linear dynamics model for each node in the network such that the time evolution of the system is given by


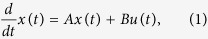


where 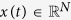 with each component *x*_*i*_(*t*) representing the state of node *i* at time *t*, 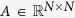 describes the interconnection of the nodes and is the transpose of the adjacency matrix of the network, 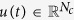 is the collection of external driving inputs to the system at time *t*, and 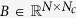 describes the assignment of controls *u*_*j*_(*t*), *j* = 1,…, *N*_*c*_, to nodes (i.e., where the controls are applyed to the network). These systems are studied in the generic sense, conceptually equivalent to considering a network without regard for the weights of the edges. Assessing structural, or generic, controllability is significantly more computationally tractable and it also focuses effort on properties most relevant to networks - their structure. Specifically, in the absence of a predefined *B*, the maximum matching algorithm can be used to identify a *B* such that the pair [*A B*] is controllable^3^,^19^.

Liu *et al*. have demonstrated several fundamental properties of controllability of networks which revolve around the fact that the number of controls required to guarantee controllability is relatively invariant under a degree-preserving shuffle (i.e., the network connections are randomized with the constraint that the degree distribution must remain the same)^1^,^20^. Posfai *et al*. identified the role that in-out degree correlations play in explaining the observed error that exists in the correlation between degree distribution and number of controls required^21^. Jia *et al*. introduced a classification that labels nodes according to whether they must, can, or are never directly connected to the minimal set of controls required to fully control the network^18^.

The current authors created a framework to cluster and classify networks based on the functional origin of their control structures^2^. This framework facilitates not only a simpler estimation method for the number of controls needed, but also provides insight as to the reasons behind why a specific node must be controlled. Our prior work identifies that the number of nodes that require direct control include source nodes (nodes with only outgoing edges), excess sink nodes (nodes with only incoming edges, but only if there are more sink nodes than source nodes), and internal dilation points (points that create the need for an additional control, but not related to source or sink nodes). For the purposes of this paper this is most easily expressed as


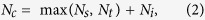


where *N*_*c*_ is the minimal control count, *N*_*s*_ is the number of source nodes, *N*_*t*_ is the number of sink nodes, and *N*_*i*_ is the number of internal dilations. An isolated node (a node without any edges) is considered as both a source and a sink node. This prior work also found that the generative algorithms which construct the most popular synthetic networks create networks which have systemically fewer internal dilations when compared to real world networks. It was shown that by counting sources and sinks alone, *N*_*c*_ could be predicted nearly as well as the degree-preserving shuffle method reported by Liu *et al*.^1^.

In the remainder of this paper, we calculate the expected number of source, sink, and isolated nodes in the most popular directed synthetic network models: Erdos-Renyi (ER), Barabasi-Albert (BA), and local-attachment (LA). Combining these counts with (2) we achieve tight estimates for the number of controls required by these various network models using very little computational effort.

## Results

### Erdos-Renyi

The Erdos-Renyi generative model describes a class of networks produced by iteratively adding edges to an initially empty graph consisting of *N* nodes^16^. Each of the 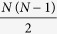 possible edges in the network is introduced with probability *p* ∈ [0, 1].

We compute the probabilities of a node in a directed ER network being a source, a sink, and an isolate. All three cases influence whether a control will be required. Similar to the undirected case described above, a directed ER network is specified using the number of nodes, *N*, and the probability of a link existing, *p* (note the number of possible edges in the directed network is *N*(*N* − 1)).

Given such a network, the probability that a node is a source is the probability that it has no inbound edges, (1 − *p*)^*N*−1^, and at least one edge outbound from it, (1 − (1 − *p*)^*N*−1^). This yields the expression


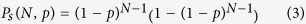


Thus, the expected number of sources in the ER network is


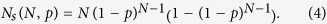


The probability of a node being a sink is the probability that no edge is outbound to it and that it has at least one edge inbound to it. Notice that, by the symmetry of the Erdos-Renyi generative process, these probabilities are the same as for source nodes. Thus, we have that *N*_*t*_(*N, p*) = *N*_*s*_(*N, p*).

Finally, the probability that a node is an isolate is the probability that it received no inbound or outbound links. So the number of isolates is


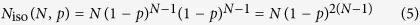


Since an isolate is simultaneously a source and sink node, *N*_iso_ is added to both *N*_*s*_ and *N*_*t*_. The estimated expected number of controls required for an ER network generated with parameters (*N, p*) is given by (2) assuming *N*_*i*_ ≈ 0, i.e.,


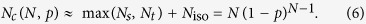


**Figure 1:**
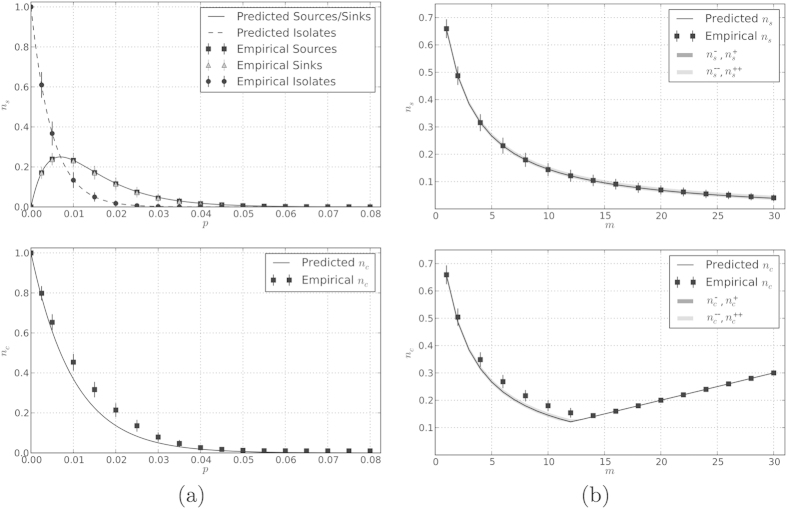
Estimated expected and empirical fractions of source, sink, and isolated nodes as well as controlled nodes (*n*_*c*_ = *N*_*c*_/*N*) in (a) Erdos-Renyi and (b) Barabasi-Albert type networks are estimated by closed form expressions; *N* = 100. Error bars represent an empirical survey over 1000 random instances of each (*N, p*) or (*N, m*) pair.

The agreement of our estimates for fractions of source, sink, and isolated nodes as well as the predicted fraction of minimum controls required for the network can be seen in Fig. 1(a).

In the large *N* limit, we can simplify *n*_*c*_ = *N*_*c*_/*N* further, using 
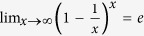
 and the average degree, *p* = *k*/(*N* − 1), to yield


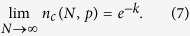


Because we explicitly omit *N*_*i*_ > 0 from this estimate, it is a lower bound for the expected fraction of controls in an ER network. This is corroborated by a similar observation that in the large *k* limit *n*_*c*_ ~ *e*^−*k*/2^, based on calculations from the cavity method^1^. Many networks of importance are sparse (negating a large *k* assumption), so our method provides a useful analytic lower bound on the fraction of nodes which must be controlled in an ER network. Moreover, the gap between these results begins to shed light on the expected number of internal dilations (*N*_*i*_) present in ER networks.

### Barabasi-Albert

The Barabasi-Albert network model is among the most widely known methods for generating networks with a scale-free degree distribution. Under this method, a network is constructed by iteratively adding nodes which, at the time of introduction, select *m* outbound neighbors. An existing node’s probability of being selected as a neighbor of node *i* is proportional to the number of edges incident to it, typically expressed as 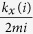, where *k*_*x*_(*i*) is the degree of node *x* at time *i* (the time step coinciding with the index of the node being added). This process is continued until a specific network size (i.e., number of nodes) is reached^15^.

In this section, we investigate a standard directed version of the BA algorithm. To bootstrap the process, *m* unlinked nodes are constructed and then one node is introduced with outbound links to the first *m* nodes. Since all nodes added subsequently have *m* outbound neighbors, no new sinks or isolates will be created. Thus, in a BA network, there are *m* sinks and no isolated nodes. Worth noting is that the preferential factor, 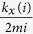, remains unchanged - in that the total degree of a node determines the probability with which it receives an edge.

To compute the number of sources, we must determine the probability that a node *x* receives no new incident edges after joining the network. Observe that such a node will have *k*_*x*_(*j*) = *m* for all time steps *j*, since it creates *m* outbound edges when first created. Thus, the probability of a source node *not* receiving an edge in time step *j* is


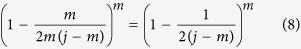


where the term (*j* − *m*) accounts for the fact that the first *m* nodes introduced into the network created no edges (hence making them sinks).

The probability that node *i* is still a source at time *N* is the product


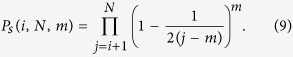


By summing *P*_*s*_ across all of the nodes after the bootstrapping phase the expected number of source nodes is given by


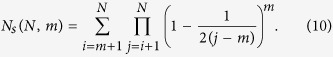


Notice that when *i* = *N* the product indexes from *j* = *N* + 1 to *j* = *N*. This notation is well-defined and called the empty product, taking the value of 1. This is consistent since the *N*^th^ node is a source node with probability 1, since it is the last node added and only has edges leaving it. Next, we adjust the indices of the summation to yield a simpler expression,


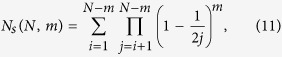


and rearrange the interior,


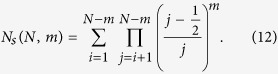


The interior product is a truncated ratio of factorials. Using the generalized factorial, Gamma function,


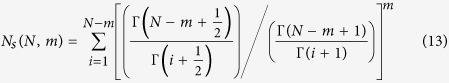


or more simply,


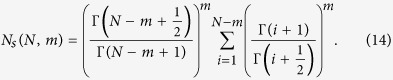


Several results exist on bounds of Gamma ratios separated by 1/2^22^. While tighter bounds exist, the following result is a useful bound, which has a compact expression but still performs well^23^,


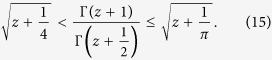


Making use of the previous result gives


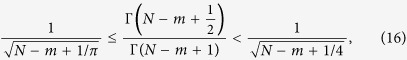


for the outer ratio and


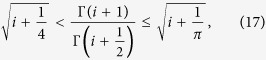


for the inner ratio. Therefore, the estimated number of source nodes in a Barabasi-Albert network is approximated by the bounds, 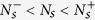, where these bounds are given by


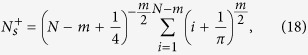



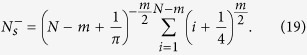


As the size of the network gets large, we can approximate the summation with an integral. Because *N*_*s*_ is a decreasing function, the integral will necessarily underestimate the sum. If we, however, shift the limits of integration to the right, from [1, *N* − *m*] to [2, *N* − *m* + 1], the integral will consistently overestimate the sum of a decreasing function. Therefore, 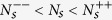, where these bounds are given by


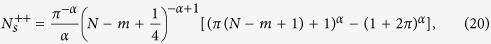



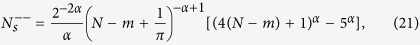


where we have introduced *α* = (*m* + 2)/2 to simplify these expressions. The estimated number of controls required for a BA network is then the maximum between any of these estimations and the number of sinks in the network, *m*. For large average degrees relative to the size of the network, sinks eventually begin to dominate sources as can be seen in Fig. 1(b).

It is worth noting that the number of sinks present in the network is entirely a product of the seed network used. Crucially, were a fully connected clique of *m* (or even 2) nodes used, there would be no sinks at all. Regardless, since the number of sinks (or sources or isolates) contributed by the seed network is fixed and, generally, much smaller than the size of the final network, the contribution of the seed network to controllability properties is both trivial and often insignificant.

### Local Attachment

Local attachment is another mechanism which creates networks with scale-free degree distributions. Similar to the BA method, nodes are introduced incrementally and select an outbound neighborhood of fixed size, *m*. Unlike the BA method, node degree does not explicitly influence the probability of inclusion in a new node’s neighborhood. Instead, neighbors are selected in a two-stage process. First, during the *random attachment phase, m*_*r*_ nodes are selected with uniform probability to be neighbors. Then, during the *local attachment phase*, the remaining *m*_*n*_ = *m* − *m*_*r*_ neighbors are selected (again with uniform probability) from among the outbound neighbors of the *m*_*r*_ nodes chosen during the first phase. The network is bootstrapped by seeding it with a clique of *m* + 1 nodes (thus, each node in the clique has *m* outbound neighbors)^17^.

By virtue of the starting condition, there are no sinks in a local attachment network. Furthermore, there are no isolates since every node is born with *m* outbound edges. From a controllability perspective, the question, then, is how many sources should be expected in a network of *N* nodes who have formed *m*_*r*_ random neighbors and *m*_*n*_ network neighbors such that *m* = *m*_*r*_ + *m*_*n*_?

For a node to cease being a source, it must receive at least one edge. Crucially, the first edge that a node receives must be formed through random attachment, since the node at that time would have no inbound neighbors that would mediate a neighbor-based interaction. Thus, the probability that node *i* is a source is the probability that it never received a random edge from nodes *i* + 1 ... *N*


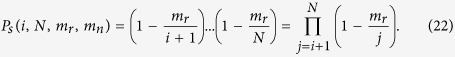


This can be rewritten using the generalized Gamma function, as


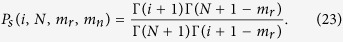


Note that *i* > *m* + 1. The expected number of sources (and an estimate of the minimal control count), then, is


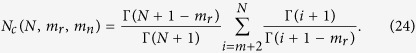


We can make use of the following result from fractional calculus to simplify this expression^24^


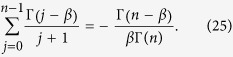


In order to achieve this form, we reindex (24) with *j* = *i* − *m*_*r*_.


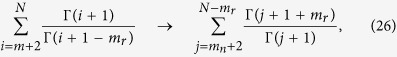


recalling that *m* − *m*_*r*_ = *m*_*n*_. The summation can be written as a difference of summations,


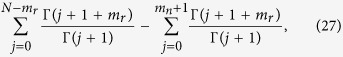


which applying (25) reduces to


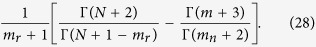


Replacing the summation in (24) with this last result, we find that the expected number of source nodes in a local attachment network is given by


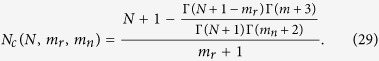


Much as with the Barabasi-Albert model, the choice of seed network can introduce some fixed number of (or exclude the option for) source, sink, or isolated nodes. For instance, in the seed network used for our implementation of LA, no sources, sinks, or isolated nodes are created. A seed more similar to that used by the BA model would produce additional sinks. However, while seeds can change the absolute number of structures relevant to control, for large networks the number of seed-contributed structures is insignificant compared to those generated by the generative process itself. Thus, for any sufficiently large network, the contribution of the seed network to the minimal control count is inconsequential.

**Figure 2:**
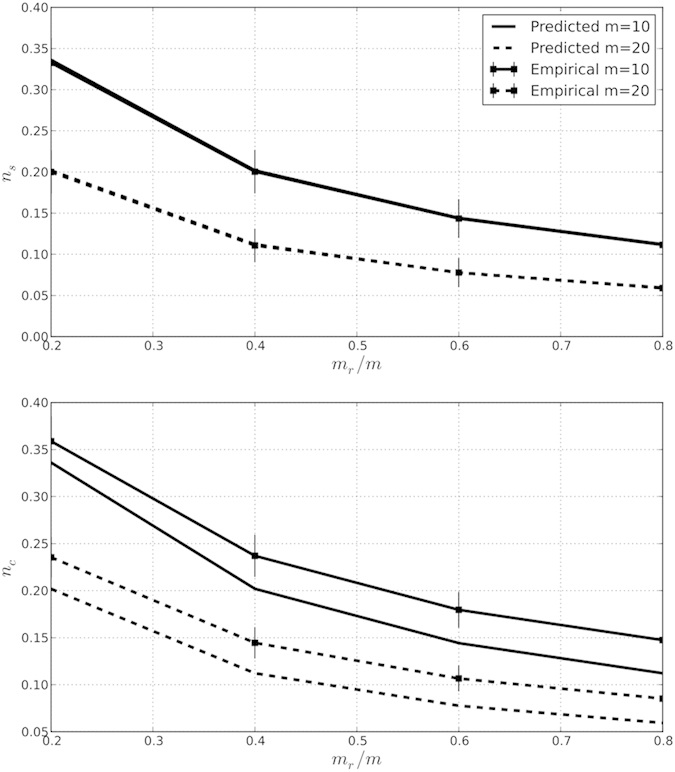
Estimated expected and empirical fractions of source nodes (*n*_*s*_ = *N*_*s*_/*N*) and controlled nodes (*n*_*c*_ = *N*_*c*_/*N*) in local attachment type networks are estimated by closed form expressions; *N* = 100. Error bars represent an empirical survey over 1000 random instances of each (*N, m, m*_*r*_) triple.

As shown in Fig. 2, our analytic expression shows excellent agreement with the observed number of sources in local attachment networks, generated across a range of parameter values. Shown are network ensembles generated for two choices of out-degree (*m*) with the involvement of the local attachment mechanism ranging from dominant (*m*_*r*_ = 0.2) to small (*m*_*r*_ = 0.8). Parameter values do not appear to impact the overall fit.

This parameter independence also extends to using the analytic expressions to estimate the minimal control count. The second panel of Fig. 2 shows that, while some discrepancy exists (a difference of <5%), the analytic results follow the actual MCC closely and, moreover, statistically bounds the true MCC from below.

Notice that changing *m*_*r*_/*m* alters the proportion of neighbors chosen with uniform probability. This has the effect of increasing or decreasing the contribution of local attachment involved in the formation of the networks, specifically tuning the power-law parameters *γ* = *m*_*r*_/*m*_*n*_ + 2^17^. The second panel in Fig. 2 reveals that, as the contribution of local attachment decreases (i.e., *m*_*r*_/*m* → 1), the number of controls falls. This inverse relationship between the number of controls and *m*_*r*_/*m* (the proportion of connections made by random attachment) is related to the finding reported by Liu *et al*., that as *γ* → 2, 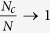; in the case we consider here, we see that as *m*_*r*_/*m* decreases (i.e., *γ* approaches 2) 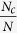 markedly increases (eventually approaching 1). Our approximation explains why this is the case: it clearly follows from Equation 22 that a smaller *m*_*r*_ makes (1 − *m*_*r*_/*j*) larger, resulting in more controls due to source nodes.

## Discussion

Since studies of real-world networks often involve comparisons with null models in order to establish the statistical significance of key features, the foremost contribution of the present work is a set of analytic expressions which provide a good estimate of the minimal control count of networks belonging to three classes of network null models commonly used in literature.

The expressions derived provide the expected number of controls induced by all structures (sources, sinks, and isolates) except for internal dilations. As a result, the expressions provide a kind of statistical lower bound for the number of controls required by a network — in the sense that these synthetic networks have some small number of internal dilations as well.

To quantify this, we ran empirical tests over a wide range of parameter values with the number of nodes ranging from *N* = 100 to *N* = 50000 and average degree ranging from *k* = 2 to *k* = 58 for all three network types. For LA networks, four values of *m*_*r*_/*m* were chosen at each *N* and *k*. For each set of parameter values, ten networks were created and evaluated to yield an average *N*_*c*_ value. The approximations provided by our expressions reproduce the value of *N*_*c*_ with small error. When the error (*N*_*c*_ − max(*N*_*s*_, *N* − _*t*_)) is calculated relative to the number of nodes in the network (quantifying the fraction of nodes correctly identified as controls), we obtain average error rates of 0.5% (ER), 2.5% (BA), and 1.9% (LA). When the error is calculated relative to the number of actual controls (quantifying the fraction of controls correctly identified), we obtain average error rates of 4.3% (ER), 18.1% (BA), and 4.3% (LA). Thus, as results from simulations show, the number of internal dilations is systematically quite small compared to the number of sources and sinks.

Often it is desirable to be able to tune particular features in order to make null network models either similar to or different from real-world networks along those particular dimensions. Future studies will seek to set the MCC of null network models.

Our analytic expressions deliver unfortunate news where this “tuning” is concerned: the parameters used to specify each network model cannot be used to modify the number of sources, sinks, or isolates in generated networks. As a result, the present network models quite definitively lack the ability to tune or set the MCC to a desired value.

While in this paper we have only shown this limitation for the three models considered, we anticipate that this is a limitation of most, if not all, existing random network models. This suggests an important direction for future work in this area: the development of derivative network models in which the minimum control count, and even the *components* of that count, can be explicitly set using model parameters.

In addition to a general lack of ability to tune controllable properties, all three models shared in common the fact that the greatest deviation from the analytic estimates derived occurred at intermediate values of parameters. In all three cases, the intermediate values correspond to networks with a moderate degree of connectivity.

This phenomena offers some insight into pre-conditions for internal dilations — the only control-inducing structure that is not accounted for by our expressions. The higher error rate indicates that a higher proportion of dilations occur at intermediate levels of connectivity.

In very well-connected networks, the probability of directed paths that traverse large sets of nodes are high. Thus, any possible dilation structures are rendered irrelevant by paths of influence that span large sections of the network.

In the case of sparse networks, we conjecture that at least two related phenomena depress the number of internal dilations. First, a paucity of edges increases the likelihood that any given node is either a source or a sink — in which case they would belong to structures estimated by our expressions. Second, internal dilations involve relatively complicated structures which will occur more rarely when few edges are available.

Overall, we consider this analytic approach to understanding control structures of random null models to be a pursuit which will yield practical tools for network analysis as well as deeper insights into the structural basis for control in complex systems.

## Additional Information

**How to cite this article**: Ruths, D. and Ruths, J. Estimating the Minimum Control Count of Random Network Models. *Sci. Rep.* **6**, 19818; doi: 10.1038/srep19818 (2016).
